# Effects of magnesium and potassium supplementation on insomnia and sleep hormones in patients with diabetes mellitus

**DOI:** 10.3389/fendo.2024.1370733

**Published:** 2024-10-29

**Authors:** Sidra Khalid, Shahid Bashir, Riffat Mehboob, Tehreem Anwar, Muhammad Ali, Mariam Hashim, Humaira Waseem, Shahnai Basharat

**Affiliations:** ^1^ Institute of Diet and Nutritional Sciences, Faculty of Allied Health Sciences, The University of Lahore, Lahore, Pakistan; ^2^ Lahore Medical Research Center, Lahore, Pakistan; ^3^ University Institute of Food Science and Technology, Faculty of Allied Health Sciences, The University of Lahore, Lahore, Pakistan; ^4^ Fatima Jinnah Medical University, Lahore, Pakistan

**Keywords:** diabetes mellitus, insomnia severity index, serum cortisol, serum melatonin, serum magnesium, serum potassium

## Abstract

**Objectives:**

Diabetes mellitus is a metabolic condition with hyperglycemia. Literature has shown a correlation between poor sleep quality and duration with an increased incidence of insomnia in diabetic individuals. The goal of this study was to determine the magnesium and potassium supplementation effect among diabetic individuals with insomnia.

**Methods:**

A randomized controlled trial (single blind) was conducted on 320 patients with diabetes; after 2 months of follow-up, 290 patients completed the trial. The Insomnia Severity Index (ISI) was used to assess the severity and duration of insomnia, before and after the trial. Tablets containing supplements were prepared: placebo (T1), magnesium (Mg, T2), potassium (K, T3), and a combination of Mg and K (T4). Melatonin and cortisol (sleep hormones) were measured from blood (serum) using an enzyme-linked immunosorbent assay (ELISA), before and after the trial.

**Results:**

The study included 93 (32.1%) male and 197 (67.9%) female participants. According to the analysis, there was a significant association between the treatment groups and ISI after the trial (post-trial), *p* = 0.0001. Analysis showed that there was significant association between pre- and post-serum cortisol levels in treatment groups 2, 3, and 4 (T2, T3, and T4) as *p*-values are 0.001, 0.001, and 0.001 respectively. Similar findings were observed for serum melatonin.

**Conclusions:**

The study revealed that magnesium, potassium, and magnesium and potassium combined had a significant effect on serum cortisol and melatonin levels (sleep hormones). In addition, supplementation significantly decreased the severity of insomnia among patients with diabetes by improving sleep duration.

## 1 Introduction

Diabetes mellitus is a metabolic disorder known for consistently high levels of glucose in the blood. It is one of the metabolic disorders that affect individuals worldwide (1). Diabetes mellitus might occur when pancreatic beta cells are unable to secrete any or very little insulin, due to insulin insensitivity in the body. There are three widely known types of diabetes mellitus, namely, type 1 diabetes, type 2 diabetes, and gestational diabetes. Apart from these three types, there are two other rare types of diabetes: secondary diabetes and monogenic diabetes (2). Type 1 diabetes usually occurs due to genetic disorders, autoimmune dysfunction, or environmental factors such as toxins and viral infections. It is a major incident among children and young adults, but it can occur at any age. The most common type of diabetes mellitus is type 2. It is prevalent among 90% of the diabetic population (3, 4). According to the International Diabetes Foundation (IDF), there are 81% of people worldwide who remain undiagnosed for diabetes, while the burden of diabetes lies more in developing countries accommodating 75% of the total population with diabetes (5). Because of the multiple medical effects and related issues, people with diabetes have an inadequate standard of living (6). It is hardly unexpected that such individuals have much worse sleeping conditions. Because of the physiological imbalances and associated issues with sleep, people with diabetes may have trouble falling asleep and staying awake (7).

Sleep is a naturally habitual condition of mind and body. It is associated with tainted consciousness, altered or reduced sensory activity, diminished muscle movement, reticence of all voluntary muscles, and decreased interaction with the surroundings. All living species on Earth, animals, insects, human, etc., exhibit sleep as a common behavior (8, 9). Worldwide, approximately 15% to 20% of individuals suffer from chronic insomnia, which is demonstrated as having insomnia for more than 1 month persistently, and apart from this percentage, another one-third of the population suffers from transient insomnia (10). Difficulty in sleeping and compromised sleep quality can worsen symptoms of diabetes. Several studies found a direct relationship between poor sleep quality and the quantity with the onset of insomnia in patients with diabetes (11). Magnesium exists as the fourth most profuse cation in the human body and the second most abundant intracellular cation. Magnesium is capable of inducing deep sleep and also acts as a muscle relaxant. A lifestyle causing irregular circadian rhythms results in the excretion of magnesium from the body resulting in magnesium deficiency (12). A change in magnesium status is often seen in patients with type 2 diabetes mellitus. Patients with diabetes type 2 have been shown to have a higher frequency of magnesium deficiencies, particularly in those with poorly managed glycemic profiles, longer illness durations, and the presence of chronic micro- and macrovascular problems (13). Potassium, the most abundant intracellular cation, plays an important role in the cellular function of nerve and muscle tissue. Clinical practice often observes potassium deficiency or dyskinesia. Electrolyte abnormalities, especially hypo- and hyperkalemia, are of critical importance because both can lead to severe or life-threatening cardiac arrhythmias and even death, especially in patients with cardiovascular or renal disease (14).

Insomnia is a growing concern in the world population, along with its association with inadequate diet patterns leading to ignorance of important nutrients. This study aimed to evaluate the effects of magnesium and potassium supplementation on diabetic patients with insomnia. This study hypothesizes that magnesium and potassium supplements can potentially reduce the intensity and duration of insomnia. The findings of this research will provide deeper insight into the relationship between insomnia and micronutrient deficiency and help reduce the burden of this disease on society.

## 2 Materials and methods

A randomized controlled trial (single blind) was conducted by using a non-probability purposive sampling technique. Trial was registered at clinicaltrial.gov, and the trial number is NCT04642313. Ethical approval (IRB-UOL-FAHS/760/2020) was obtained from the Institutional Review Board (IRB) of the Faculty of Allied Health Sciences (FAHS), The University of Lahore. Patients with diabetes having insomnia according to ISI were enrolled in the study, and their pre- and post-trial blood samples were collected. Prior written informed consents were taken from the entire study participants; they were made aware of the study purpose. All the participants were free to leave the trial at any stage at their own will. The study duration was 24 months, November 2020 to November 2022. The proposed place of work was the Diabetes Center of Akhuwat Health Services, Lahore. The sample size was calculated by the following formula:


$$n_{1}=\frac{{\left(z_{1-\frac{\alpha }{2}}+z_{1-\beta }\right)}^{2}\left[\sigma_{1}^{2}+\frac{\sigma_{2}^{2}}{r}\right]}{{\text{Δ}}^{2}}$$

$$r=\frac{n_{2}}{n_{1}},\text{Δ}=\mu_{1}-\mu_{2}$$

*z*_1–β_ is the desired power of study = 95%

*z*_1-α/2_ is the desired level of significance at 5%

*μ*_1_ is the mean in group 1 of insomnia severity (mean of Mg intake group) = 14.14 (15)

*μ*_2_ is the mean in group 2 of insomnia severity (mean of the placebo group) = 15.77 (15)

*σ*_1_ is the standard deviation (SD) in group 1 of insomnia severity (SD of Mg intake group) = 2.68 (15)

*σ*_2_ is the standard deviation (SD) in group 2 of insomnia severity (SD of the placebo group) = 1.92 (15)

Ratio (*r*) = 1

Alpha (*α*) = 0.05

Beta (*β*) = 0.2

*n* (expected sample size) = 66 in each group (×4)

**Figure 1:**
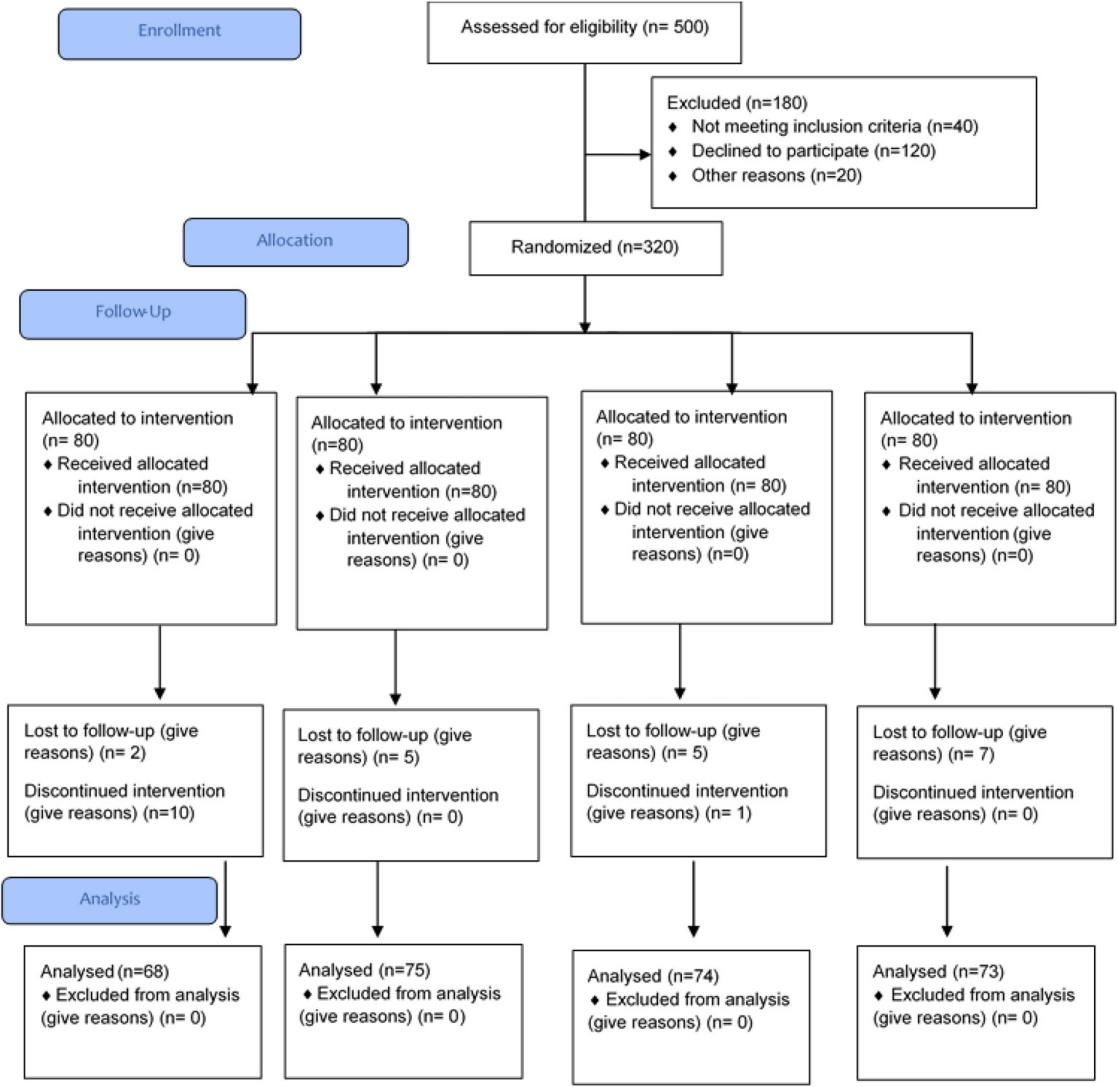
Consort flow diagram.

The sample size was adjusted according to the 20% dropout rate. The expected sample was 80 in each treatment group, as there were four treatment groups; the total sample size was 320 with 20% dropout. A total of 320 patients were selected and followed up, but after 2 months, 290 patients were followed up and completed the trial. The study included patients with diabetes of both genders aged between 19 and 65 years with insomnia history and without conditions like hypomagnesemia and hypokalemia. Diabetic patients with certain medical conditions such as insomnia due to psychological reasons, hormonal therapy, cardiovascular diseases, severe liver injury or severe cirrhosis, kidney diseases, drug-induced insomnia, sleep disorders, anxiety, restless leg syndrome, sleep deprivation, and alcohol consumption were not included. Treatment tablets were prepared at Paragon Laboratories and FORM-6 was also acquired from these (as per the guidelines of the Drug Regulatory Authority of Pakistan, Pakistan Act 1976). Tablets including placebo (T1), magnesium (Mg, T2), potassium (K, T3), and a combination of Mg and K (T4) were prepared; each tablet contained 250 mg of the desired treatment. The placebo tablets contained starch. Mg tablets were made using magnesium gluconate salt; for potassium tablets, potassium chloride salt was used, and for the Mg+K group, both of these salts were used. Child-safe bottle packing for tablets was used. Tablets were stored in a cool dry place and were provided to patients every month according to the doses shown in **Figure 1**. Helsinki guidelines were followed during the trial. Treatment groups were assigned randomly to the study participants by the physician, and it was ensured that blood samples were taken under fasting conditions. Blood samples (before and after) were collected by a qualified phlebotomist, for sleep hormones (melatonin and cortisol) and serum electrolytes. Sleep hormones such as melatonin and cortisol (AM, morning) were measured from blood (serum) using a quantitative and sensitive enzyme-labeled immunosorbent assay (ELISA). For melatonin, the Human MT (Melatonin) ELISA Kit (Cat. No. E-EL-H2016, Elabscience) was used, and for cortisol, the Human Cortisol ELISA Kit (Cat. No. E-EL-0157; Elabscience) was used.

Along with the assigned treatment tablets, study participants were given an eating plan with magnesium and potassium under 75% RDA, with dietary guidelines designed according to Pakistan dietary guidelines. Statistical analysis was performed using SPSS version 25.0. Numerical data including age and hormonal levels were presented as mean ± SD. The chi-square test was used to analyze categorical data, and statistical significance was set at *p*-value ≤ 0.05. The H-test was used to determine whether there was a significant difference in the post-ISI scores between the groups, and statistical significance was measured at *p*-value ≤ 0.05.

## 3 Results

The mean age (± SD) of study participants suffering from diabetes mellitus with insomnia is shown in **Table 1**.

**Table 1:** Mean age (years) of patients with diabetes suffering from insomnia within treatment groups.

Age (years)	Treatment groups			
	T1	T2	T3	T4
**Mean ± SD**	48 ± 9	51 ± 9	50 ± 11	48 ± 9

T1: Placebo (starch tablets 250 mg × 2).

T2: Magnesium (250 mg × 2).

T3: Potassium (250 mg × 2).

T4: Magnesium + potassium (250 mg × 2).

**Figure 2:**
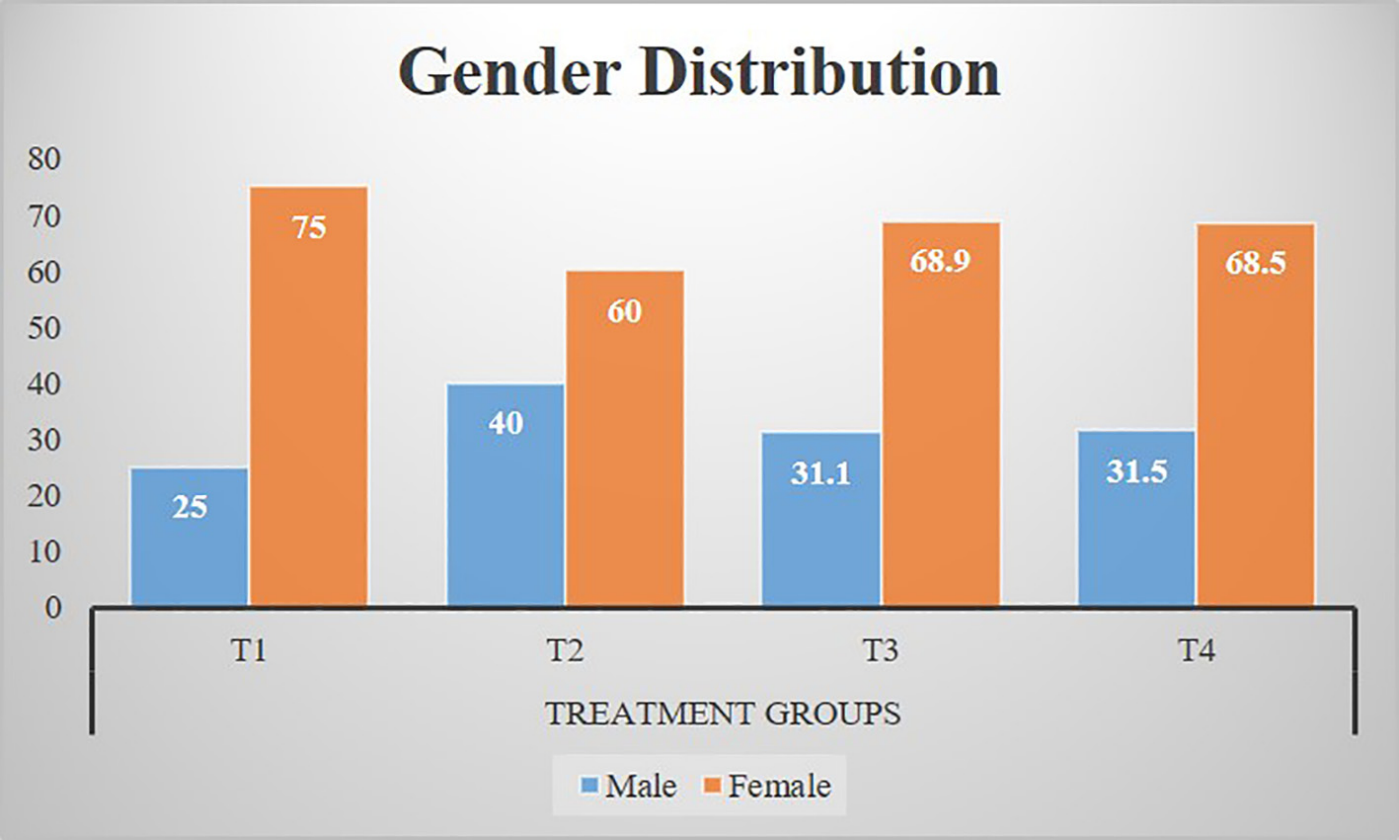
Gender distribution of participants among treatment groups. T1: Placebo (starch tablets 250 mg × 2). T2: Magnesium (250 mg × 2). T3: Potassium (250 mg × 2). T4: Magnesium + potassium (250 mg × 2).

**Figure 2** shows that there were 93 (32.1%) male and 197 (67.9%) female participants in the study. Moreover, **Figure 2** also describes the gender-wise distribution of study participants in treatment groups.

**Table 2** shows the pre- and post-serum cortisol levels (μg/dL) between groups. Analysis showed that there was a significant association between pre- and post-serum cortisol levels in treatment groups 2, 3, and 4 (T2, T3, and T4) as *p*-values are 0.001, 0.001, and 0.001 respectively.

**Table 2:** Mean serum cortisol levels (μg/dL) pre- and post-trial among diabetic patients with insomnia within treatment groups.

Serum cortisol level (μg/dL)		T1	T2	T3	T4
**Difference (mean** ± **SD)**	**Pre-trial**	42.55 ± 9.08	40.71 ± 9.36	41.44 ± 9.29	41.57 ± 10.01
	**Post-trial**	40.75 ± 8.46	24.75 ± 6.41	31.69 ± 8.39	22.70 ± 4.07
***p*-value**		0.247	0.03	0.04	0.001

T1: Placebo (starch tablets 250 mg × 2).

T2: Magnesium (250 mg × 2).

T3: Potassium (250 mg × 2).

T4: Magnesium + potassium (250 mg × 2).

**Table 3** shows a comparison of pre- and post-trial cortisol levels (μg/dL) between treatment groups according to the Kruskal–Wallis *H* test.

**Table 3:** Comparison of pre- and post-trial cortisol levels (μg/dL) between treatment groups.

Variable	T1	T2	T3	T4	Chi-square	*p*-value
**Pre-trial cortisol (mean rank)**	154.22	138.26	145.88	144.64	1.302	*0.792*
**Post-trial cortisol (mean rank)**	233.43	104.77	167.72	84.67	135.74	0.000*

*p-value was obtained by the Kruskal–Wallis H test with <0.05 level of significance. Data presented in mean rank.

T1: Placebo (starch tablets 250 mg × 2).

T2: Magnesium (250 mg × 2).

T3: Potassium (250 mg × 2).

T4: Magnesium + potassium (250 mg × 2).

**Table 4** shows the pre- and post-serum melatonin levels (pg/mL) between groups. Analysis showed that there was a significant association between pre- and post-serum melatonin levels in treatment groups 2, 3, and 4 (T2, T3, and T4) as *p*-values are 0.001, 0.001, and 0.001 respectively.

**Table 4:** Mean serum melatonin levels (pg/mL) pre- and post-trial among diabetic patients with insomnia within treatment groups.

Serum melatonin level (pg/mL)		T1	T2	T3	T4
**Difference (mean** ± **SD)**	**Pre-trial**	7.03 ± 2.32	5.79 ± 2.43	6.26 ± 1.90	6.12 ± 2.25
	**Post-trial**	6.84 ± 1.61	6.17 ± 2.07	6.50 ± 1.78	15.37 ± 17.37
***p*-value**		0.502	0.047	0.189	0.001

T1: Placebo (starch tablets 250 mg × 2).

T2: Magnesium (250 mg × 2).

T3: Potassium (250 mg × 2).

T4: Magnesium + potassium (250 mg × 2).

**Table 5** shows a comparison of pre- and post-trial melatonin levels (pg/mL) between treatment groups according to the Kruskal–Wallis *H* test.

**Table 5:** Comparison of pre- and post-trial melatonin levels (pg/mL) between treatment groups.

Variable	T1	T2	T3	T4	Chi-square	*p-*value
**Pre-trial melatonin (mean rank)**	170.70	135.57	142.12	136.13	8.22	*0.140*
**Post-trial melatonin (mean rank)**	157.10	111.97	144.17	171.11	20.7	0.000*

*p-value was obtained by the Kruskal–Wallis H test with <0.05 level of significance. Data presented in mean rank.

T1: Placebo (starch tablets 250 mg × 2).

T2: Magnesium (250 mg × 2).

T3: Potassium (250 mg × 2).

T4: Magnesium + potassium (250 mg × 2).

Analysis showed that there was a significant association between the treatment groups and Insomnia Severity Index (ISI) at baseline (pre-trial), *p* = 0.0001, as shown in **Table 6**.

**Table 6:** Distribution and comparison of diabetic patients with insomnia according to Insomnia Severity Index (ISI) categories within treatment groups (at baseline).

ISI categories	Treatment groups				Total	*p*-value
	T1	T2	T3	T4		
**No clinically significant insomnia**	0 (0.0%)	1 (1.3%)	1 (1.4%)	4 (5.5%)	6 (2.1%)	0.0001
**Sub-threshold insomnia**	3 (4.4%)	17 (22.7%)	20 (27.0%)	4 (5.5%)	44 (15.2%)	
**Clinical insomnia (moderate severity)**	42 (61.8%)	42 (56.0%)	45 (60.8%)	45 (61.6%)	174 (60.0%)	
**Clinical insomnia (severe)**	23 (33.8%)	15 (20.0%)	8 (10.8%)	20 (27.4%)	66 (22.8%)	
**Total**	68 (100.0%)	75 (100.0%)	74 (100.0%)	73 (100.0%)	290 (100.0%)	

T1: Placebo (starch tablets 250 mg × 2).

T2: Magnesium (250 mg × 2).

T3: Potassium (250 mg × 2).

T4: Magnesium + potassium (250 mg × 2).

According to the analysis, there was a significant association between the treatment groups and ISI after the trial (post-trial), *p* = 0.0001, as shown in **Table 7**.

**Table 7:** Distribution and comparison of diabetic patients with insomnia according to Insomnia Severity Index (ISI) categories within treatment groups (after the trial).

ISI categories	Treatment groups				Total	*p*-value
	T1	T2	T3	T4		
**No clinically significant insomnia**	0 (0.0%)	23 (30.7%)	24 (32.4%)	30 (41.1%)	77 (26.6%)	0.0001
**Sub-threshold insomnia**	19 (27.9%)	22 (29.3%)	21 (28.4%)	21 (28.8%)	83 (28.6%)	
**Clinical insomnia (moderate severity)**	22 (32.4%	30 (40.0%)	29 (39.2%)	22 (30.1%)	103 (35.5%)	
**Clinical insomnia (severe)**	27 (39.7%)	0 (0.0%)	0 (0.0%)	0 (0.0%)	27 (9.3%)	
**Total**	68 (100.0%)	75 (100.0%)	74 (100.0%)	73 (100.0%)	290 (100.0%)	

T1: Placebo (starch tablets 250 mg × 2).

T2: Magnesium (250 mg × 2).

T3: Potassium (250 mg × 2).

T4: Magnesium + potassium (250 mg × 2).

**Table 8** shows a comparison of post-treatment ISI category scores between treatment groups according to the Kruskal–Wallis *H* test.

**Table 8:** Comparison of post-treatment Insomnia Severity Index (ISI) category scores between treatment groups.

Variable	T1	T2	T3	T4	Chi-square	*p*-value
**Pre-trial ISI score (mean rank)**	145.18	148.61	129.78	158.49	4.481	*0.214*
**Post-trial ISI score (mean rank)**	160.68	140.24	149.36	133.12	4.27	0.233

*p-value was obtained by the Kruskal–Wallis H test with <0.05 level of significance. Data presented in mean rank.

T1: Placebo (starch tablets 250 mg × 2).

T2: Magnesium (250 mg × 2).

T3: Potassium (250 mg × 2).

T4: Magnesium + potassium (250 mg × 2).

**Figure 3:**
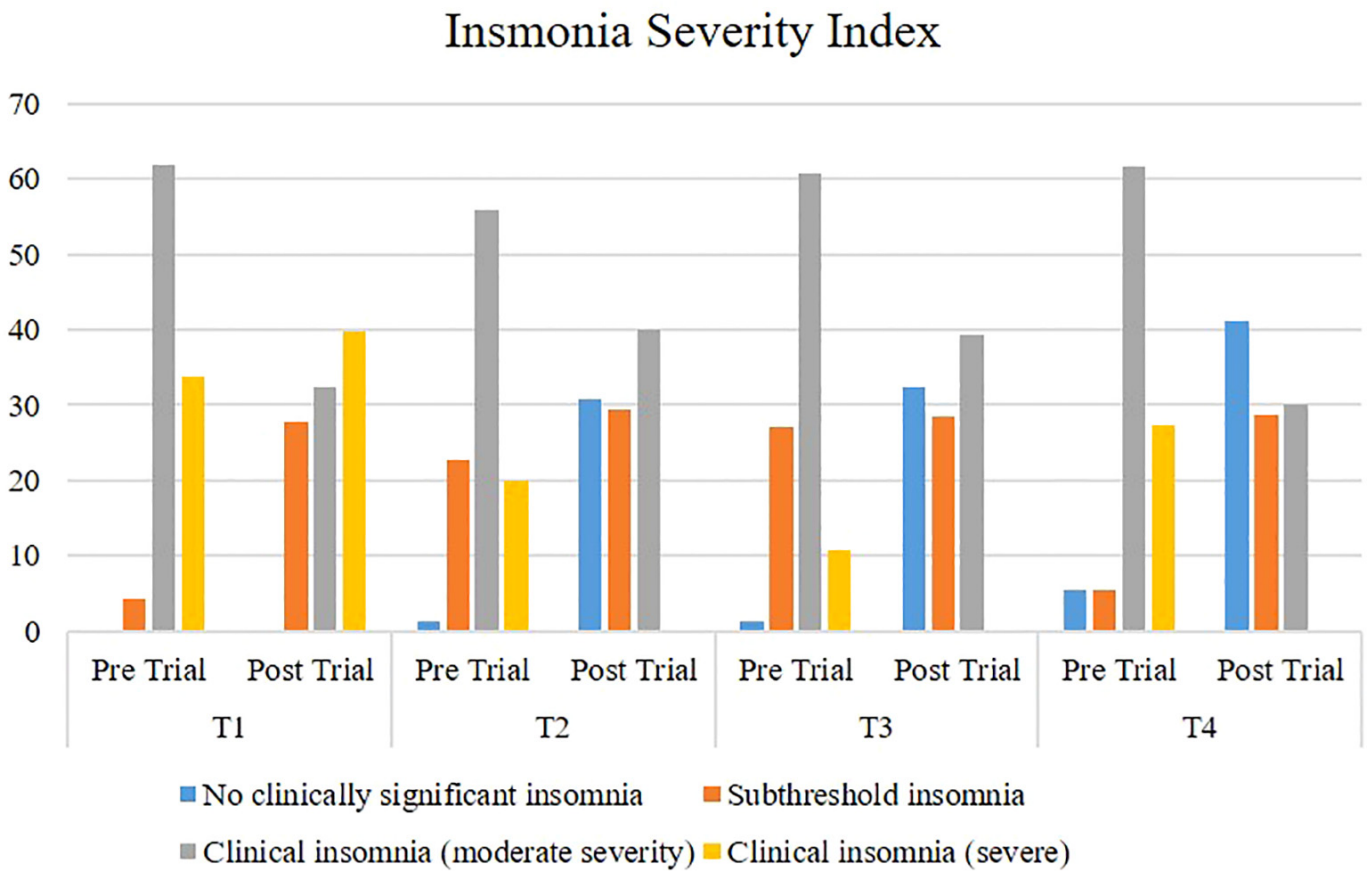
Comparison of Insomnia Severity Index among participants pre- and post-trial. T1: Placebo (starch tablets 250 mg × 2). T2: Magnesium (250 mg × 2). T3: Potassium (250 mg × 2). T4: Magnesium + potassium (250 mg × 2).

**Figure 3** shows a comparison of ISI among participants before and after the trial. In the placebo group post-trial, there was a decrease in participants with moderate clinical insomnia; in the T2 (magnesium) group, participants with moderate and severe clinical insomnia were significantly reduced post-trial. In the T3 (potassium) group, a significant decrease was noted in participants with moderate and severe clinical insomnia, and in the T4 (Mg + K) group, a significant reduction was reported in participants with severe and moderate clinical insomnia after the trial. However, there was an increase in participants with sub-threshold insomnia in all groups post-trial.

## 4 Discussion

According to results, in the placebo group, the mean age was 48 ± 9 years, whereas according to Jahrami et al., the mean age of participants in the placebo group was 43 ± 15 years, and it was 65.4 ± 4.5 years according to Abbasi et al. (15, 16). In the study by LeBlanc et al., the mean age of diabetic patients with insomnia in the placebo group was 57.1 (± 13.8) years (17). In the current study, the mean age of participants in the magnesium group was 51 ± 9 years, whereas according to the study by Abbasi and colleagues on insomnia and magnesium supplementation, the mean age of participants receiving magnesium supplements was 64.7 ± 4.7 years (15). Analysis revealed that more than half (two-thirds) of the participants were women, which explains that the number of female participants suffering from insomnia due to diabetes is double than that of male participants in the study population. In contrast, according to the findings of a similar study (selected as parent study) conducted by Abbasi and colleagues to determine the effect of magnesium on elderly people with insomnia, the ratio of male and female participants was equal (15). Quite comparable findings were also noted by Jahrami et al., who reported to have 61% of female participants in their study while they were investigating the association between micronutrients and sleep quality (16). In another study by LeBlanc et al., they reported to have a near-equal ratio of male (46.4%) and female (53.6%) participants while finding the association between diabetes and insomnia, which is not in accordance with the current findings (17), while the findings of Cao and colleagues support the findings of the current study. Cao et al. determined the relation between magnesium intake and insomnia, and they also clearly reported to have a 2:3 ratio of female to male participants in their trial (18).

Analysis showed a significant association between the treatment groups and ISI score, *p* = 0.0001. Abbasi et al. (15) also reported that magnesium supplementation importantly reduced ISI ratings (*p* = 0.006), which supports the current findings (15). Our results are corroborated by a meta-analysis of experimental and observational research, which found that the amount of sleep has a negative correlation with the levels of vitamin B12, Cu, K, and Zn and a positive correlation with the levels of Fe, Zn, and Mg (19). Held et al. assessed the effects of magnesium medication for 20 days on the phases of sleep in 12 older persons in a placebo-controlled randomized crossover research; however, they used different sleep assessment techniques. Their results corroborated current research findings since it was shown that taking magnesium supplements caused a considerable elevation in slow-wave (deep) sleep (20). The present research results were also supported by a study on patients with insomnia, which found that supplementing with magnesium, melatonin, and vitamin B complex for 3 months was useful in treating insomnia of any kind, independent of the underlying reason (21). According to a meta-analysis by Arab et al., observational studies have established a relationship between magnesium levels and sleep quality, including daytime sleepiness, overall sleepiness, and sleep duration. However, the association between magnesium supplementation and sleep disorders remains questionable despite a review of all available randomized clinical trials (22). A case–control study of healthy and depressed patients by Jahrami et al. revealed that the micronutrient status of vitamin B12 and Mg can predict sleep quality in healthy controls. Our study also found that sleep quality was positively correlated with Mg intake, thus providing additional evidence to the existing literature (16). Contrary to the current study findings, no associations were found between dietary magnesium intake and daytime sleep (18).

In addition, magnesium supplementation importantly reduced ISI ratings (*p* = 0.006) (15). A comparison between pre-treatment mean ± SD of T4 (magnesium + potassium group) and post-treatment mean ± SD showed a significant association within groups as the *p*-value is 0.00. Abbasi and colleagues worked on magnesium supplementation and revealed that dietary magnesium improved serum melatonin level (*p* = 0.007) (15). Khalid and coworkers conducted a similar study on magnesium and potassium supplementation among diabetic patients with insomnia and concluded that the supplementation had no significant effect on the quality of life of the study participants (23).

## 5 Conclusion

This study revealed that magnesium, potassium, and magnesium and potassium combined had a significant effect on serum cortisol and melatonin levels (sleep hormones), whereas the placebo group had an insignificant association with sleep hormones, but still showed some improvement in the level and severity of insomnia among patients. In addition, supplementation significantly decreased the severity of insomnia among patients with diabetes.

## 6 Strengths of the study

- No previous study has targeted insomnia among people suffering from diabetes mellitus.
- Both magnesium and potassium were examined together with different treatment groups among diabetic patients with insomnia.

## 7 Limitations of the study

There were certain limitations of this study that should be considered while doing further research:

- The study was conducted in only one region of Lahore, Punjab, Pakistan, and a specific group from almost the same socioeconomic status was targeted.
- All patients with diabetes were selected regardless of duration of being diabetic.
- Patients were not screened for depression or anxiety.
- Responses from different people vary, making generalization difficult. Other factors include compliance as well as adhesion problems, risk factors associated with elevated dosage medications, drug interactions, unclear ideal amount, eating habit uncertainties, the necessity to take immediate as well as long-lasting consequences into account, and a small sample size.

## Data Availability

The raw data supporting the conclusions of this article will be made available by the authors, without undue reservation.
